# Evaluation of a maternity waiting home and community education program in two districts of Malawi

**DOI:** 10.1186/s12884-018-2084-7

**Published:** 2018-11-23

**Authors:** Kavita Singh, Ilene S. Speizer, Eunsoo Timothy Kim, Clara Lemani, Jennifer H. Tang, Ann Phoya

**Affiliations:** 1 Department of Maternal and Child Health, Gillings School of Global Public Health, University of North Carolina at Chapel Hill, Chapel Hill, NC USA; 2 Carolina Population Center, University of North Carolina at Chapel Hill, Chapel Hill, NC USA; 3 UNC Project-Malawi, c/o Kamuzu Central Hospital, 100 Mzimba Road, Private Bag, A-104 Lilongwe, Malawi; 4 Department of Obstetrics & Gynecology, School of Medicine, University of North Carolina at Chapel Hill, Chapel Hill, NC USA

**Keywords:** Maternity waiting homes, Community education, Evaluation, Maternal health, Malawi

## Abstract

**Background:**

The implementation of Maternity Waiting Homes (MWHs) is a strategy to bring vulnerable women close to a health facility towards the end of their pregnancies. To date, while MWHs are a popular strategy, there is limited evidence on the role that MWHs play in reaching women most in need. This paper contributes to this topic by examining whether two program-supported MWHs in Malawi are reaching women in need and if there are changes in women reached over time.

**Methods:**

Two rounds of exit interviews (2015 and 2017) were conducted with women within 3 months of delivery and included both MWH users and non-MWH users. These exit interviews included questions on sociodemographic factors, obstetric risk factors and use of health services. Bivariate statistics were used to compare MWH users and non-MWH users at baseline and endline and over time. Multivariable logistic regression was used to determine what factors were associated with MWH use, and Poisson regression was used to study factors associated with HIV knowledge. Descriptive data from discharge surveys were used to examine satisfaction with the MWH structure and environment over time.

**Results:**

Primiparous women were more likely to use a MWH compared to women of parity 2 (*p* < 0.05). Women who were told they were at risk of a complication were more likely to use a MWH compared to those who were not told they were at risk (*p* < 0.05). There were also significant findings for wealth and time to a facility, with poorer women and those who lived further from a facility being more likely to use a MWH. Attendance at a community event was associated with greater knowledge of HIV (*p* < 0.05).

**Conclusions:**

MWHs have a role to play in efforts to improve maternal health and reduce maternal mortality. Education provided within the MWHs and through community outreach can improve knowledge of important health topics. Malawi and other low and middle income countries must ensure that health facilities affiliated with the MWHs offer high quality services.

**Electronic supplementary material:**

The online version of this article (10.1186/s12884-018-2084-7) contains supplementary material, which is available to authorized users.

## Background

Millennium Development Goal 5 (MDG 5), a three quarters reduction in maternal mortality from 1990 to 2015, was an elusive goal for the majority of low and middle income countries. Only nine such countries achieved this goal. Though Sustainable Development Goal (SDG) 3 is broadly focused on health for all, the emphasis on maternal health has not diminished. The first SDG 3 target is to reduce the global maternal mortality ratio to less than 70 maternal deaths per 100,000 live births by 2030. According to 2015 estimates, the global maternal mortality ratio stands at 216 maternal deaths per 100,000 live births [1]. For this global SDG target to be met, substantial efforts are needed to improve maternal health and reduce maternal mortality. Access to maternal health care, including a facility that provides basic emergency obstetric and newborn care (EmONC), is key. Though the majority of pregnancies proceed normally, complications can arise without warning, and some can be fatal if prompt treatment is not available. Improved access to maternal care for vulnerable women and high quality care for all women will be needed to reach the first SDG 3 target [2].

Maternity waiting homes (MWHs) are typically buildings close to a health facility or rooms within a health facility. Pregnant women who have complications or who live far from a health facility are often encouraged to stay in them towards the end of their pregnancy [3–5]. The intention is to increase timely access to maternal care for women who are vulnerable because of heightened risk of complications or long distance. Studies of MWHs have looked at issues such as quality of care and barriers to use. In a qualitative thematic analysis of 19 studies, Penn-Kekana and colleagues [6] identified four key factors to be considered when implementing MWHs: 1) community engagement; 2) quality, cleanliness and safety of MWH structure and environment; 3) quality of services provided at the corresponding health facility; and 4) financial and operational sustainability of the MWH. Studies from Guatemala and Sierra Leone have found community engagement and family support to be essential for increasing the use of MWHs [7, 8]. Henry and colleagues [9] examined both the quality of the MWH (or waiting space within a health facility) and the ability of the corresponding health facility to provide basic EmONC. The quality of the MWH was based upon a composite measure, which included factors related to the physical structure of the facility or waiting space, water source, toilet facility, bedding and cooking facilities. Facility delivery coverage was higher in catchment areas with a medium or high quality MWH, in contrast to catchment areas with a low quality MWH (64% versus 49%). The study also found that women in catchment areas with a MWH had a 19% increase in the odds of facility delivery compared to women in areas without a MWH, after controlling for capacity of the health facility to provide EmONC and demographic factors.

The World Health Organization’s Recommendations on Health Promotion Interventions for Maternal and Newborn Health [10] include MWHs as a conditional recommendation due to limited evidence on their effectiveness. This conditional recommendation specifically states that, “MWHs are recommended to be established close to a health facility where essential childbirth care and/or care for obstetric and newborn complications is provided to increase access to skilled care for populations living in remote areas or with limited access to services.” In terms of sustainability, many MWHs are established by governments, donors or non-governmental organizations, but community support is vital in terms of set-up, maintenance and provision of food and transport [6, 8, 11, 12].

A limited number of studies have looked at risk factors of MWH users compared to MWH non-users in terms of distance, previous pregnancy complications, poverty and parity. Most of the studies that have looked at distance have found that MWH users come from further distances than non-users [13–17]. In contrast, a study from Timor Leste found that facility delivery coverage did not increase for women living greater than 25 km from a health facility after the introduction of MWHs [18]. Based upon a cross-sectional survey in Ethiopia, Vermeiden et al. [19] found that intention to use a MWH was higher among women who had a previous pregnancy complication compared to those that did not. A study in Zambia found that MWH users were more likely to have had complications during the antenatal care period than non-MWH users [16]. In Malawi, MWH users were more likely to have a prior negative pregnancy outcome than non-MWH users [17]. Researchers in Tanzania, however, found that obstetric risk defined as being of nulliparous status (having a first birth) or grand multipara status (greater than a 4th birth), having a previous cesarean section and poor obstetric history was not associated with MWH use [15]. Poverty has been found to be associated with MWH use in Ethiopia [13], Tanzania [16] and Malawi [17]. Higher parity compared to primigravida status was found to be associated with MWH use in Ethiopia [13], while another study in Ethiopia found parity to be nonsignificant [19]. MWH users at a district hospital in Malawi were more likely to be of nulliparous or of parity four or higher compared to non-MWH users, though the differences were not significant [17].

In Malawi, maternal mortality fell 34% from 957 maternal deaths per 100,000 live births in 1990 to 439 maternal deaths per 100,000 live births in 2015 (MDHS 2015/2016) [1]. The Presidential Initiative on Maternal Health and Safe Motherhood was launched in 2012 in an effort to accelerate reductions in maternal and neonatal mortality. There are three elements to this initiative 1) community mobilization and training of local leaders, 2) construction of maternity waiting homes, and 3) training of community midwives. As part of this initiative, UNC Project-Malawi supported the development of two MWHs, trained community midwives and integrated quality improvement processes in maternity services. In addition, the project supported the training of local leaders about the importance of maternal health, family planning and HIV prevention. Community mobilizations occurred after the training of local leaders. The MWHs provided women a safe place to stay towards the end of their pregnancy and daily educational sessions on maternal and child health topics, including HIV prevention. The HIV prevalence for Malawian women ages 15–49 is 11.2% [20]. This paper presents findings from the evaluation of UNC Project’s two supported MWHs and corresponding programs. The primary objectives are to examine if there are changes in the characteristics of MWH users and non-MWH users over time and to determine which characteristics are associated with MWH use. Key variables of interest included risk factors for pregnancy complications, wealth and time to the facility. Secondary objectives are to understand whether HIV knowledge differs between MWH users and non-MWH users and to present data on satisfaction with the MWH structure and environment over time.

## Methods

### Study setting

UNC Project constructed two MWHs and conducted education in their surrounding communities on the importance of maternal health, HIV prevention and family planning services through its Safe Motherhood Initiative. The initiative officially started in May 2013 and ended in December 2017. The first MWH was constructed at a semi urban health center, the Area 25 Health Center, which had opened in October 2014. The second MWH was constructed at Kasungu District Hospital; it opened as a temporary structure in 2012 and was replaced by a permanent structure in December 2015. The two settings are quite different as Area 25 is located in Lilongwe, the capital city of Malawi, while Kasungu is in the center of a rural district. Patient loads also vary given that Area 25 is a primary-level health center and Kasungu is a secondary-level District Hospital, which receives patient referrals from health centers across the district. Also important to note are the differences in the provision of food to patients. As a health center, Area 25 does not provide food to any of its inpatients, including MWH users, whereas Kasungu District Hospital provides food to all inpatients including MWH users.

### Exit interviews

Two rounds of exit interviews were conducted with both MWH users and non-MWH users at the Area 25 Health Center and Kasungu District Hospital. A total of 553 interviews were conducted at baseline, and 639 were conducted at endline. (The exit interview questionnaire is presented as Additional file 1.) The baseline or first round was conducted from April to July 2015, and the endline was conducted from June to September 2017. It should be noted that the first round of exit interviews was not a true baseline because support to the MWHs had already begun. The first round, thus, reflects early program implementation and results can be found in Singh et al. [18]. The target sample for the exit interviews included both MWH users and non-MWH users who just recently delivered, were visiting for postnatal care check (within 6 weeks of delivery) or who were bringing in a child < 3 months for a wellness visit. This latter group was asked whether they had used the MWH for the delivery of their most recent baby and were meant to capture a larger sample of MWH and non-MWH users. At the time of each survey, women were asked if they had already been interviewed recently at the facility. This was meant to avoid interviewing a woman twice in a round of data collection. The number of clients interviewed by location and round are presented in Table 1. The number of MWH-users interviewed was higher in Kasungu than Area 25 because of the higher client load at Kasungu. The exit interview questions were focused on sociodemographic characteristics, knowledge of maternal and child health, use of the MWHs and maternal health services, birth outcomes and family planning use and intentions to use.

**Table 1:** Number of Exit Interviews

	Baseline	Endline
	April – July 2015	June – September 2017
Area 25 MWH	87	137
Area 25 non-MWH	150	181
Kasungu MWH	175	150
Kasungu non-MWH	141	171

### Statistical analyses for the exit interviews

Stata version 14 software (StataCorp, College Station, TX, USA) was used to conduct the analyses. Exit interview data were first compared by χ^2^ test and *t* test to determine if MWH users and non-users by facility differed in terms of key socioeconomic and pregnancy-related factors. For each facility, comparisons were made between MWH users and non-MWH users at both baseline and endline, and comparisons were also made among MWH users and non-MWH users over time. The covariates that were studied included age, education, husband’s education, parity, marital status, prior pregnancy outcome and being told that one is at risk of complications. Household factors included a measure of wealth (based on the quality of the toilet type) and self-reported time to the health facility. Exposure to community education events among MWH users and non-users was also studied as a programmatic factor. Based on the literature, the key risk factors for maternal morbidity and mortality (and thus for being recommended to stay at a MWH) were low or high parity, being poor, prior negative pregnancy outcome and distance or time to the facility. We chose to look at time to facility rather than distance, because time would account for travel method and terrain.

Multivariable logistic regression was used to understand which factors were associated with MWH use. Poisson regression was used to understand whether MWH use was associated with HIV knowledge, after controlling for other key factors. Due to the required model assumption that the variance should be equal to the mean, a negative binomial regression was also run as a potential alternative because this model relaxes that assumption. The results of the negative binomial model, as well as the deviance goodness-of-fit test and the Pearson goodness-of-fit test, all indicated that running a Poisson model was appropriate and that its results were no different than the results of the negative binomial model. Hence, we kept the Poisson model. The HIV knowledge outcome was an additive index with a score ranging from 0 to 4 based on responses to questions on ways HIV can be transmitted to a newborn (during pregnancy, during delivery and through breastfeeding) and on whether there are medicines to prevent newborns from getting HIV.

### Descriptive data from discharge surveys

In Area 25, to supplement the exit interviews, intake and discharge surveys were used to capture information regarding MWH users. These surveys included questions on socio-demographic characteristics, decision-making, reasons for MWH use, guardians (person staying with the pregnant woman at the MWH) and satisfaction with the MWH structure and environment. In this paper, we use data from 457 discharge surveys to provide descriptive data over time on satisfaction with the Area 25 MWH, including location of the MWH within the facility, noise level, cleanliness, kitchen and toilet facilities and sense of personal safety. Women were asked to rate the MWH on these characteristics using a Likert scale with the categories of poor, fair, good, very good and excellent. (The discharge questionnaire is available as Additional file 2.)

### Ethics approval

This study was approved by the Institutional Review Board at the University of North Carolina at Chapel Hill and the National Health Science Research Committee in Malawi. Oral consent was obtained before the exit interviews with women, which was deemed appropriate for the study setting where low literacy is an issue. The discharge data were part of routine care and did not require consent procedures in accordance with the ethics review boards.

## Results

Table 2 presents results comparing MWH users and non-users at both baseline and endline and also over time for Area 25. About 42% of women in the baseline sample were age 20–24 years, and 22% were age 25–29 years. Similarly, at endline, 38% were age 20–24 years, and 24% were age 25–29 years. There were no significant differences in age between MWH users and non-MWH users, nor were there significant differences over time. The majority of women had some formal education and for those women who were married, the majority of their husbands had some formal education. There were significant differences between MWH users and non-users at endline in terms of marital status with 79% of MWH users compared to 98% of MWH non-users being married (*p* < 0.001). The change in percentage of MWH users who were currently married from baseline (95%) to endline (79%) was also significant (*p* = 0.001).

**Table 2:** Comparisons between MWH users and non-MWH users at baseline and endline and comparisons of changes over time at Area 25

	Baseline				Endline					
	MWH (%)	Non-MWH (%)	Total (%)	Difference between MWH users and non-users (*p* value)	MWH (%)	Non-MWH (%)	Total (%)	Difference between MWH users and non-users (*p* value)	Change in MWH users over time (*p* value)	Change in non-MWH users over time (*p* value)
Sociodemographic Factors										
Age group				0.751				0.847	0.634	0.524
15–19	18.6	15.4	16.6		17.9	20.0	19.1			
20–24	44.2	40.3	41.7		37.4	37.7	37.5			
25–29	20.9	22.2	21.7		26.8	22.4	24.2			
30–34	8.1	13.4	11.5		12.2	15.3	14.0			
35+	8.1	8.7	8.5		5.7	4.7	5.1			
Education level				0.578				0.352	0.23	0.244
None	1.2	2.0	1.7		3.0	5.5	4.5			
Primary	58.6	52.0	54.4		47.7	51.9	50.2			
Secondary or higher	40.2	46.0	43.9		49.2	42.5	45.4			
Marital Status				0.428				< 0.001*	0.001*	0.788
Currently not married	4.6	2.7	3.4		20.6	2.2	10.1			
Currently married	95.4	97.3	96.6		79.4	97.8	89.9			
Husband’s Education				0.478				0.397	0.100	0.481
None	0	1.4	0.9		2.0	3.5	3.0			
Primary	33.3	29.2	30.7		21.4	27.5	25.3			
Secondary or higher	66.7	69.4	68.4		76.5	69.0	71.8			
Key Risk Factors										
Parity				0.165				0.045*	0.625	0.025*
1	42.5	31.3	35.5		48.9	38.1	42.8			
2–4	49.4	55.6	53.3		43.1	56.9	50.9			
5+	8.1	13.2	11.3		8.0	5.0	6.3			
Prior Birth Outcome				0.025*				0.369	0.550	0.355
Live birth	76.6	90.5	85.9		81.3	86.4	84.5			
Adverse outcome	23.4	9.5	14.1		18.7	13.6	15.5			
Told Could be at Risk for Complications				0.832				< 0.001*	0.54	0.002*
Yes	54.7	56.1	55.6		58.8	38.7	47.3			
No	45.3	43.9	44.4		41.2	61.3	52.7			
Type of toilet				< 0.001*				< 0.001*	0.003*	< 0.001*
Not in house	46.5	4.7	19.9		66.9	25.4	43.2			
In house	53.5	95.3	80.1		33.1	74.6	56.8			
Mean time to health facility	50.6 ± 31.8	43.3 ± 30.8	46.2 ± 31.3	0.092	46.4 ± 36.9	58.2 ± 56.5	53.2 ± 49.5	0.043*	0.397	0.007*
Programmatic Factor										
Attend community event				0.932				0.70	0.001*	0.031*
No	80.5	80.0	80.2		59.8	69.6	65.4			
Yes	19.5	20.0	19.8		40.2	30.4	34.6			
Number of observations	86	149	235		123	170	293			

*p < 0.050

At endline, MWH users and non-users differed significantly in terms of parity. There were more women having either a first (49% versus 38%) or fifth or higher order (8% versus 5%) pregnancy among MWH users compared to non-users, respectively (*p* = 0.045). In terms of prior birth outcomes, at baseline significantly more MWH users (23%) compared to non-MWH users (10%) had a previous adverse prior birth outcome (*p* = 0.025). There was no difference in being told that one was at risk of complications between MWH users (55%) and non-MWH users (56%) at baseline; however, the differences in being told that one was at risk for complications at endline were statistically significant (59% for MWH users and 39% for non-MWH users, *p* = 0.001). At endline the difference between MWH users (19%) and non-MWH users (14%) in reporting of prior adverse pregnancy outcomes was not significant.

There were significant differences between MWH users and non-MWH users in socioeconomic status. At both baseline and endline, significantly fewer MWH users than non-MWH users had a toilet inside their home (*p* < 0.001 for both). The decreases in the percentages of both MWH users and non-MWH users who had a toilet inside their home over time were also significant (*p* = 0.003 and *p* < 0.001, respectively). Mean time to facility was greater for MWH users (51 min) than non-MWH users (43 min) at baseline (but only at *p* = 0.092), but at endine, this reversed and mean time to facility was greater for non-MWH users (58 min) compared to MWH users (46 min), which was a significant difference (*p* = 0.043). Attendance at community events was significantly higher at endline compared to baseline for both MWH users and non-MWH users (*p* = 0.001 and *p* = 0.031, respectively). At baseline 20% of MWH users attended community events compared to 60% at endline (*p* = 0.001), while 20% of non-MWH reported attendance at baseline and 70% at endline (*p* = 0.031).

Table 3 contains data for Kasungu and describes the same variables as presented in Table 2 for Area 25. Thirty-four percent of women at baseline were age 20–24 years, followed by 22% age 15–19 years. At endline, 34% were age 20–24 years, and 30% were age 15–19 years, which was not significantly different from baseline. The majority of respondents were currently married, and the majority of both respondents and their husbands’ had some formal education. There were no significant differences between MWH users and non-MWH users or differences over time for parity. Likewise, there were no significant differences for the prior birth outcome variable. The majority of women also had a toilet in their home. Mean time to facility was significantly higher at both baseline (*p* < 0.001) and endline (*p* < 0.001) for MWH users compared to non-users. The mean time to facility was 132 min versus 87 min at baseline for MWH-users and non-MWH users, respectively. At endline, the mean time was 121 min for MWH users and 77 min for non-MWH users. Comparing baseline to endline, attendance at community events was significantly higher at baseline for both MWH (56 to 33%, *p* < 0.001) and non-MWH users (53 to 29%, *p* < 0.001).

**Table 3:** Comparisons between MWH users and non-MWH users at baseline and endline and comparisons of changes over time at Kasungu

	Baseline				Endline					
	MWH (%)	Non-MWH (%)	Total (%)	Difference between MWH users and non-users (*p* value)	MWH (%)	Non-MWH (%)	Total (%)	Difference between MWH users and non-users (*p* value)	Change in MWH users over time (*p* value)	Change in non-MWH users over time (*p* value)
Sociodemographic Factors										
Age group				0.061				0.849	0.176	0.078
15–19	26.6	15.9	21.9		30.6	29.5	30.0			
20–24	30.6	39.1	34.4		31.9	34.9	33.6			
25–29	16.2	21.0	18.3		18.8	17.5	18.1			
30–34	11.6	14.5	12.9		12.5	9.6	11.0			
35+	15.0	9.4	12.5		6.3	8.4	7.4			
Education level				0.206				0.052	0.797	0.310
None	4.6	8.6	6.4		6.0	5.3	5.6			
Primary	68.0	60.0	64.4		68.7	56.7	62.3			
Secondary or higher	27.4	31.4	29.2		25.3	38.0	32.1			
Marital Status				0.487				0.089	0.568	0.123
Currently not married	4.0	5.7	4.8		5.3	10.5	8.1			
Currently married	96.0	94.3	95.2		94.7	89.5	91.9			
Husband’s Education				0.286				0.052	0.251	0.018*
None	4.2	2.3	3.4		7.2	6.7	6.9			
Primary	53.6	46.9	50.7		45.3	32.0	38.4			
Secondary or higher	42.3	50.8	46.0		47.5	61.3	54.7			
Key Risk Factors										
Parity				0.517				0.671	0.959	0.468
1	46.7	42.1	44.6		45.3	49.1	47.4			
2–4	37.1	43.6	40.1		38.7	38.0	38.3			
5+	16.2	14.3	15.3		16.0	12.9	14.3			
Prior Birth Outcome				0.125				0.902	0.737	0.183
Live birth	88.2	94.9	91.5		89.9	89.3	89.6			
Adverse outcome	11.8	5.1	8.5		10.1	10.7	10.4			
Told Could be at Risk for Complications				0.216				0.078	< 0.001*	< 0.001*
Yes	76.9	70.7	74.1		56.1	46.2	50.8			
No	23.1	29.3	25.9		43.9	53.8	49.2			
Type of toilet				0.955				0.023*	0.179	0.010*
Not in house	3.4	3.6	3.5		6.7	14.6	10.9			
In house	96.6	96.4	96.5		93.3	85.4	89.1			
Mean time to health facility	131.9 ± 90.3	87.0 ± 76.1	111.3 ± 86.9	< 0.001*	121.0 ± 67.6	76.7 ± 60.4	96.6 ± 67.3	< 0.001*	0.267	0.208
Programmatic Factor										
Attend community event				0.533				0.365	< 0.001*	< 0.001*
No	44.0	47.5	45.6		66.7	71.4	69.2			
Yes	56.0	52.5	54.4		33.3	28.7	30.8			
Number of observations	175	140	315		150	171	321			

*p < 0.050

Table 4 presents data on the two outcome variables. Forty-six percent of the total sample were MWH users and 54% were non-MWH users; at baseline, 47% were MWH users, and at endline 45% were the same. At Area 25, there was a greater proportion of the sample that were MWH users at endline; however, this difference was not significant. For Kasungu, there were significantly fewer MWH users surveyed at endline (47%) compared to baseline (55%, *p* ≤ 0.05). Forty-six percent of respondents were knowledgeable on three out of the four HIV questions at both baseline and endline. At baseline, only 8% of interviewed women were knowledgeable about all of the HIV facts whereas by endline this percentage had doubled to 16% (*p* ≤ 0.001). Significant increases were observed in pregnancy knowledge in both Area 25 and Kasungu (*p* ≤ 0.001 for both).

**Table 4:** Outcome variables for multivariate analyses by area of study, Malawi

	Full sample		Area 25		Kasungu	
	Baseline	Endline	Baseline	Endline	Baseline	Endline
Use of MWH (%)						
No	52.6	55.2	63.3	56.9	44.6	53.3
Yes	47.4	44.8	36.7	43.1	55.4	46.7*
Pregnancy Knowledge Scale^†^ (%)						
0	4.2	11.4	5.9	5.3	2.9	17.1
1	8.8	5.4	6.7	5.6	10.4	5.3
2	33.6	21.8	33.6	16.6	33.5	26.8
3	45.7	45.7	42.4	51.4	48.1	40.2
4	7.8	15.7***	11.3	21.0***	5.1	10.6***

^†^Pregnancy knowledge scale is an additive index based on responses to questions on ways HIV can be transmitted to a newborn (during pregnancy, during delivery and through breastfeeding) and on whether there are medicines to prevent newborns from getting HIV

*p ≤ 0.05; ***p ≤ 0.001

Table 5 displays the multivariable regression analysis results with MWH use as the outcome variable. Three models are presented - the full model (with data for both Area 25 and Kasungu) the Area 25 model and the Kasungu model. Women of parity one compared to the reference category of parity two were significantly more likely to use the MWH in the full sample of women (β = 0.42, *p* < 0.05) and the Area 25 sample (β = 0.74, *p* < 0.05). Women who were told they were at risk of a complication were significantly more likely to use the MWH in the full sample (β = 0.31, *p* < 0.05). Women who had a toilet in their homes were significantly less likely to use the MWH in the full sample (β = − 1.30, *p* < 0.001) and in the Area 25 sample (β = − 1.97, *p* < 0.001). There was a slight but significant association between longer time to the facility and MWH use for the full sample (β = 0.01, *p* < 0.001) and the Kasungu sample (β = 0.01, *p* < 0.001).

**Table 5:** Multivariate coefficients and 95% confidence intervals from logistic regression models of factors associated with use of a maternity waiting home (MWH) among women surveyed in 2015 and 2017 at postpartum, postnatal, or child welfare visits in two areas of Malawi

	Full sample MWH vs. Not MWH		Area 25 MWH vs. Not MWH		Kasungu MWH vs. Not MWH	
	β	95% CI	β	95% CI	β	95% CI
Sociodemographic Factors						
Age group						
15–19	− 0.04	(− 0.74; 0.65)	− 0.72	(−1.86; 0.42)	0.22	(− 0.69; 1.13)
20–24	− 0.08	(− 0.70; 0.54)	− 0.46	(− 1.47; 0.54)	0.00	(− 0.82; 0.83)
25–29	− 0.03	(− 0.63; 0.58)	− 0.37	(−1.34; 0.61)	0.09	(− 0.71; 0.89)
30–34	− 0.13	(− 0.72; 0.45)	− 0.49	(− 1.47; 0.50)	− 0.06	(− 0.82; 0.70)
35+ (ref)	–		–		–	
Education level						
None/Primary (ref)	–		–		–	
Secondary or higher	− 0.09	(− 0.36; 0.18)	− 0.15	(− 0.58; 0.27)	− 0.12	(− 0.50; 0.26)
Location						
Area 25 (ref)	–		–		–	
Kasungu	0.37	(0.09; 0.66)*	NA		NA	
Time period						
Baseline (2015 - ref)	–		–		–	
Endline (2017)	− 0.23	(− 0.48; 0.03)+	− 0.31	(− 0.73; 0.11)	− 0.15	(− 0.51; 0.20)
Key Risk Factors						
Parity						
1	0.42	(0.05; 0.80)*	0.74	(0.14; 1.33)*	0.16	(− 0.35; 0.68)
2 (ref)	–		–		–	
3–4	0.17	(− 0.24; 0.58)	0.12	(− 0.50; 0.75)	0.11	(− 0.47; 0.70)
5+	0.31	(−0.27; 0.89)	0.22	(−0.73; 1.17)	0.18	(−0.59; 0.95)
Told she may have complications						
No (ref)	–		–		–	
Yes	0.31	(0.05; 0.56)*	0.17	(−0.23; 0.57)	0.25	(−0.10; 0.60)
Prior Birth Outcome						
No prior/Live birth (ref)	–		–		–	
Adverse outcome	0.44	(−0.07; 0.95)+	0.67	(−0.03; 1.38)+	0.40	(−0.40; 1.21)
Type of toilet						
Not in house (ref)	–		–		–	
In house	−1.30	(−1.65; −0.96)***	−1.97	(−2.40; − 1.54)***	0.43	(− 0.23; 1.08)
Time to facility (continuous)	0.01	(0.00; 0.01)***	−0.00	(−0.01; 0.00)	0.01	(0.01; 0.01)***
Programmatic Factor						
Attended community event						
No (ref)	–		–		–	
Yes	0.18	(−0.08; 0.45)	0.13	(−0.31; 0.58)	0.20	(−0.16; 0.56)
Number of observations	1160		538		622	
pseudo R2	0.0794		0.1591		0.0690	

+ *p* < 0.10 *p < 0.05 **p < 0.01 ***p < 0.001

Table 6 contains the Poisson regression results for the HIV knowledge variable. For all three models, secondary or higher education was significantly associated with greater HIV knowledge (full sample: β = 0.12, *p* < 0.001; Area 25 sample: β = 0.11, *p* < 0.10; Kasungu sample: *p* < 0.05). For Area 25, women in the endline sample had significantly greater HIV knowledge than women in the baseline sample (β = 0.12, *p* < 0.05). Women of parity one (having their first child)) had significantly less HIV knowledge than women of parity two for the full sample (β = − 0.12, *p* < 0.05) and the Kasungu sample (β = − 0.16, *p* < 0.05). Attendance at a community event was significantly associated with HIV knowledge in the full sample (β = 0.10, *p* < 0.05). MWH use was not associated with pregnancy knowledge in any of the models. Interactions between MWH use and time were tested and found not to be significant.

**Table 6:** Multivariate coefficients and 95% confidence intervals from Poisson regression models of factors associated with pregnancy knowledge among women surveyed in 2015 and 2017 at postpartum, postnatal, or child welfare visits in two areas of Malawi

	Full sample Pregnancy knowledge		Area 25 Pregnancy knowledge		Kasungu Pregnancy knowledge	
	β	95% CI	β	95% CI	β	95% CI
Sociodemographic Factors						
Age group						
15–19	0.02	(−0.19; 0.22)	0.13	(− 0.18; 0.44)	−0.06	(− 0.34; 0.22)
20–24	− 0.01	(− 0.19; 0.18)	0.10	(− 0.17; 0.38)	−0.10	(− 0.34; 0.16)
25–29	0.07	(−0.11; 0.25)	0.17	(−0.10; 0.44)	0.01	(−0.23; 0.25)
30–34	0.10	(−0.07; 0.27)	0.17	(−0.10; 0.44)	0.06	(−0.17; 0.28)
35+ (ref)	–		–		–	
Education level						
None/Primary (ref)	–		–		–	
Secondary or higher	0.12	(0.04; 0.20)**	0.11	(−0.00; 0.22)+	0.13	(0.01; 0.24)*
Location						
Area 25 (ref)	–		–		–	
Kasungu	−0.13	(−0.22; − 0.05)**	NA		NA	
Time period						
Baseline (2015 - ref)	–		–		–	
Endline (2017)	0.02	(−0.06; 0.09)	0.12	(0.01; 0.23)*	−0.08	(−0.18; 0.03)
Key Risk Factors						
Parity						
1	−0.12	(−0.22; − 0.01)*	−0.09	(− 0.25; 0.07)	−0.16	(− 0.32; − 0.00)*
2 (ref)	–		–		–	
3–4	−0.06	(− 0.17; 0.06)	−0.00	(− 0.16; 0.16)	−0.13	(− 0.30; 0.05)
5+	0.01	(−0.16; 0.17)	0.11	(−0.14; 0.35)	−0.08	(− 0.31; 0.16)
Prior Birth Outcome						
No prior/Live birth (ref)	–		–		–	
Adverse outcome	−0.02	(−0.17; 0.13)	− 0.04	(− 0.23; 0.16)	0.01	(− 0.24; 0.24)
Type of toilet						
Not in house (ref)	–		–		–	
In house	0.01	(−0.10; 0.11)	0.05	(−0.08; 0.17)	−0.07	(− 0.27; 0.13)
Time to facility (continuous)	0.00	(−0.00; 0.00)	−0.00	(− 0.00; 0.00)	0.00	(− 0.00; 0.00)
Programmatic Factor						
Use of maternity waiting home						
Not a MWH user	–		–		–	
MWH user	−0.04	(−0.12; 0.03)	− 0.05	(− 0.17; 0.07)	−0.024	(− 0.13; 0.09)
Attended community event						
No (ref)	–		–		–	
Yes	0.10	(0.02; 0.18)*	0.08	(−0.03; 0.20)	0.08	(−0.03; 0.19)
Number of observations	1169		542		627	
Adjusted R2	0.0691		0.0640		0.0507	

+ *p* < 0.10 *p < 0.05 **p < 0.01 ***p < 0.001

Table 7 presents data on satisfaction with the structure and environment at the Area 25 MWH. Responses from the MWH users are averaged and presented in six-month intervals. The percentage of women giving excellent ratings increased over time, such that by the second half of 2016, over 96% of women gave excellent ratings for each of the categories.

**Table 7:** Excellent Ratings for MWH at Area 25 (Discharge Surveys)

	2015	2016	2016	2017
	(Second Half)	(First Half)	(Second Half)	(First Half)
	*N* = 114	*N* = 131	*N* = 108	*N* = 103
Factor				
Location of MWH in Health facility grounds*	92.1	95.4	99.1	99.0
Noise Level During the Day****	73.7	96.2	99.1	99.0
Noise Level at Night****	73.7	96.2	99.1	99.0
Amount of Light*	90.4	96.2	97.2	99.0
Amount of Water for Drinking/Cooking**	86.8	93.1	97.2	98.1
Amount of Water for Bathing****	62.3	80.2	96.3	98.1
Cleanliness of Toilet****	60.5	79.4	96.3	98.1
Kitchen/Food Preparation Facilities****	63.2	82.4	97.2	98.1
Sense of Personal Safety****	79.8	83.9	97.1	98.1

*p < 0.10 **p < 0.05***p < 0.001****p < 0.0001

## Discussion

Because of limited evidence, the WHO [10] has conditionally recommended the implementation of MWHs as a strategy to improve maternal health particularly for populations in remote areas or with limited access to services. This study demonstrated that two UNC Project-supported MWHs in Malawi are reaching women who are vulnerable in terms of obstetric risk factors, poverty and time to a health facility. These MWHs have the potential not just to provide a place to stay close to a health facility, but they are also providing education sessions on important maternal and child health topics. The literature indicates that quality of the MWH is an important demand-side factor in terms of MWH use [6, 9, 11], and satisfaction with the MWH structure and physical environment was high in our study.

Results from the multivariable regression analyses of MWH use indicated that vulnerable women were being reached. While previous studies have found mixed results on parity [13, 17, 19], we found that MWH users in the full sample and at Area 25 were more likely to be nulliparous (i.e. just about to have their first birth) than to be of parity 2 (our referent group). Given that women having a first birth are considered at greater risk of obstructed labor [21], this is a noteworthy finding. MWH users in the full sample were also significantly more likely to be told that they were at risk for a complication during their current pregnancy, which corroborates results found by Sialubanje et al. [16] in Zambia. Poorer women in the full sample and in the Area 25 sample were significantly more likely to be MWH-users than wealthier women, according to our measure of wealth. This finding is similar to that from other studies [13, 16, 19]. Women who had a longer time to the facility were significantly more likely to be MWH-uses in the full sample and the Kasungu sample. Other researchers have found similar results for distance or time to the facility [13–17].

MWH use was not associated with HIV knowledge in our multivariable regression results, but attendance at community events was significantly associated with HIV knowledge. It could be that both MWH users and non-MWH users had obtained important information from project-related community events and trainings. Educational and skills sessions at MWHs are still an important means of providing knowledge and answering individual questions from women. Such sessions could also improve the quality of the experience at the MWH by providing a close bond among the MWH users and also among the staff and the MWH users. A qualitative study of MWH users in Liberia found that women valued a restful and supportive environment and that loneliness can be an issue at the time of delivery [22]. A review of the determinants of satisfaction with maternal health services in low and middle income countries, indicated that women value information and counseling from providers [23].

Provision of a high-quality MWH structure and environment can be a means to increase the use of MWHs [6, 9, 11, 24]. A study in Zambia found that women viewed infrastructure, food, security and privacy as notable considerations when thinking about MWH use [11]. The discharge data from Area 25 revealed that the MWH scored very highly on these issues and improved over time. Unfortunately, this study did not assess satisfaction with midwifery care, which was found to be valued by MWH users in another study in Malawi [24]. Being treated in a courteous and empathetic manner has been shown to be the most commonly reported determinant of satisfaction by women seeking maternal health services in low and middle income countries [23].

This study has several limitations. We were not able to study the quality of services at the corresponding health facilities to which the MWHs were attached. According to the most recent Malawi Demographic and Health Survey (DHS), skilled delivery increased from 55% in 1992 to 91% in 2015/2016 [25]; however, maternal mortality remains high. Mgawadere et al. [26] stress that quality of care at health facilities in Malawi must be improved in efforts to prevent maternal deaths. Overall, authors have noted the importance of ensuring that the MWHs are attached to health facilities that provides high quality care [12, 27]. Another limitation is that there could be some bias in the results on satisfaction with the MWH structure and environment. Some women may not have been comfortable giving low ratings while they were still at the MWH. Finally, since the data available were only from repeated cross-sectional samples, it is not possible to determine causality between the Safe Motherhood Initiative and the outcomes of interest. This paper, however, demonstrates that users were coming from high-risk groups and that this continued over time in both settings.

## Conclusion

Reaching women who would otherwise not receive services is a key role that MWHs can play in Malawi and other low and middle income countries. Women who stay at a MWH can present to the corresponding health facility in a timely manner, which can be life-saving given that some pregnancy complications can be fatal if not treated promptly. Providing educational sessions and a high-quality safe environment can be a means of offering a positive pregnancy experience and encouraging more women to come to the MWHs. Overall this study finds that MWHs can play an important role in efforts to improve maternal health and reduce maternal mortality.

## Additional files

Additional file 1: Exit Interview Questionnaire. (PDF 146 kb)

Additional file 2: Discharge Questionnaire. (PDF 201 kb)

## Abbreviations

DHS: Demographic and Health Survey
EmONC: emergency obstetric and neonatal care
MDG: Millennium Development Goal
MWHs: Maternity Waiting Homes
PMTCT of HIV: preventing-mother-to-child-transmission
SDG: Sustainable Development Goal
SMI: Safe Motherhood Initiative
