# Supplementary material for: Evaluation of a maternity waiting home and community education program in two districts of Malawi
Source: BMC Pregnancy Childbirth. 2018 Nov 23;18:457. doi: 10.1186/s12884-018-2084-7 (PMC6251123; doi:10.1186/s12884-018-2084-7)
Supplement: Supplementary file 1 — Exit Interview Questionnaire. (PDF 146 kb) [file 12884_2018_2084_MOESM1_ESM.pdf]

UNC PROJECT MALAWI
UNIVERSITY OF NORTH CAROLINA (UNC)

EXIT CLIENT

QUESTIONNAIRE NUMBER: __ __

Facility:	01=Area 25	02=Kasungu
Interviewer code:		
Date of Interview: __ __/__ __/__ __ (DD/MM/YY)		

Interview start time: __ __: __ __ HH:MM (GMT) Interview end time: __ __: __ __ HH:MM (GMT)

CLINIC WHERE EXIT INTERVIEW TAKES PLACE: 1=Delivery services
2=Postnatal care services
3=Child wellness services (child ≤ 3 months)

FOR CHILD WELLNESS AND POSTNATAL CARE CLIENTS ONLY: Has she been interviewed by the Evaluation of the Safe Motherhood Project previously in the last month?

YES (IF YES, THANK THE RESPONDENT BUT DO NOT CONDUCT THE INTERVIEW.)
NO Continue with the interview

EXIT INTERVIEW WITH POST-DELIVERY, PNC AND CHILD HEALTH CLIENTS			
We would like to ask you a few questions to gain an understanding of maternal and newborn services in your area.			
	**QUESTIONS**	**CODING**	**SKIP/NOTES**
Q1	In what month and year were you born?	MONTH [__|__]<br>YEAR [__|__][__|__]	
Q2	How old were you on your last birthday?<br>COMPARE AND CORRECT Q1 IF INCONSISTENT	AGE IN COMPLETED YEARS [__|__]	
Q3	Have you ever attended school?	1=YES<br>2=NO	 2→Q5
Q4a	What is the highest level of school you have completed?	1=PRESCHOOL<br>2=PRIMARY<br>3=SECONDARY<br>4=HIGHER THAN SECONDARY<br>96=DON'T KNOW	
Q4b	What is the highest (class/form/year) you completed at that level?	CLASS/FORM/YEAR [__]__]	
Q5	What is your current working status?<br><br>PROBE. CIRCLE ALL MENTIONED	A=UNPAID FAMILY WORKER/HOUSEWIFE/AGRIC WORKER<br>B=UNEMPLOYED<br>C=SELF-EMPLOYED<br>D=EMPLOYEE-FORMAL WORK (PAID)<br>E=INFORMAL WORK (PAID)<br>F=OTHER ______________________<br>(SPECIFY)	
Q6	What is your current marital status?	1=MARRIED OR LIVING TOGETHER<br>2=DIVORCED/SEPARATED<br>3=WIDOWED<br>4=NEVER MARRIED AND NEVER LIVED TOGETHER	 2→Q10<br>3→Q10<br>4→Q10
Q7	Has your husband ever attended school?	1=YES<br>2=NO<br>96=DON'T KNOW	 2→Q9<br>96→Q9
Q8a	What is the highest level of school your husband has completed?	1=PRESCHOOL<br>2=PRIMARY<br>3=SECONDARY<br>4=HIGHER THAN SECONDARY<br>96=DON'T KNOW	
Q8b	What is the highest (class/form/year) your husband completed	CLASS/FORM/YEAR [__]__]<br>DON'T KNOW 96	
Q9	What is your husband's usual working status?<br><br>PROBE. CIRCLE ALL MENTIONED	A=UNPAID FAMILY WORKER/FARMER<br>B=UNEMPLOYED<br>C=SELF-EMPLOYED<br>D=EMPLOYEE-FORMALWORK (PAID)<br>E=INFORMAL WORK (PAID)<br>F=OTHER ________________<br>(SPECIFY)	
Q10	What is the size of your household?	________ (NUMBER)	
Q11	Who is the head of your family?	01=SELF<br>02=HUSBAND<br>03=DAUGHTER<br>04=DAUGHTER-IN-LAW<br>05=GRANDCHILD<br>06=MOTHER<br>07=MOTHER-IN-LAW<br>08=SISTER<br>09=OTHER RELATIVE<br>10=ADOPTED/FOSTER/ STEPCHILD<br>11=NOT RELATED<br>12=OTHER	

Q13	What is the main source of drinking water in your household?	1=PIPE INSIDE<br>2=PIPE OUTSIDE<br>3=TANKER<br>4=SPRING/RAIN<br>5=WELL	6=BOREHOLE<br>7=RIVER/STREAM<br>8=DUGOUT<br>9=PACKET WATER<br>10=OTHER	
Q14	What is the type of toilet in your household?	1=WATER CLOSET<br>2=PIT LATRINE IN HOUSE<br>3=KVIP IN HOUSE<br>4=BUCKET/PAN	5=PUBLIC TOILET<br>6=COMMUNAL TOILET<br>7=BUSH/FREE RANGE<br>8=OTHER	
Q15	What fuel do you mainly use for cooking?	1=WOOD<br>2=ELECTRICITY<br>3=KEROSENE/PARAFIN<br>4=CHARCOAL<br>5=ANIMAL/CROP WASTE	6=SAWDUST<br>7=LPG GAS<br>8=BIOGAS<br>9=NO FOOD COOKED IN HH<br>10=OTHER	
Q16a	How far is your household from this facility?	____________(KILOMETERS)<br>996=DON'T KNOW		
Q16b	How long did it take for you to get from your home to this facility?	TIME IN MINUTES: ________	(CONVERT TO MINUTES IF GIVEN IN HOURS)	
Q16c	What means of transport did you use to get to the facility? Was it your own or a paid vehicle (if applicable)?	1= WALK<br>2=BICYCLE (paid)<br>3=BICYCLE (personal)<br>4=MOTORCYCLE (paid)<br>5=MOTORCYCLE (personal)<br>6=BUS/TAXI/CAR (paid)<br>7=PERSONAL CAR<br>8=AMBULANCE/HEALTH FACILITY VEHICLE<br>9=OTHER ________________<br>(SPECIFY)		

PREGNANCY HISTORY			
Now I would like to ask some questions about your pregnancy and birth history.			
	**QUESTIONS**	**CODING**	**SKIP/NOTES**
Q17	What was the outcome of your recent pregnancy?	1=LIVE BIRTH<br>2=STILLBIRTH<br>3=MISCARRIAGE<br>4=LIVE BIRTH THAT DIED WITHIN 1 DAY	
Q18	Prior to your most recent pregnancy, how many previous pregnancies have you had?	_____ (RECORD NUMBER OF PREGNANCIES)	If zero, skip to Q26
Q19	What was the outcome of the next most recent pregnancy?	1=LIVE BIRTH<br>2=STILLBIRTH<br>3=MISCARRIAGE<br>4=LIVE BIRHT THAT DIED WITHIN 1 DAY	
Q20	Between the end of your next most recent pregnancy (live birth or another outcome) and the delivery for this recent pregnancy, how many months passed?	_____ (RECORD NUMBER OF MONTHS)	
Q21	How many live born children have you ever given birth to (including this birth)?	_____ (RECORD NUMBER OF LIVE BIRTHS)	If “none” skip to Q26
Q22	Sometimes bad things happen, have you ever had any of your live born children die?	1=YES<br>2=NO<br>96=PREFER NOT TO ANSWER	If No or prefer not to answer, skip to Q26
Q23	How many children have died?	________ (NUMBER)	
Q24	Have you ever had any live born children die within the first year of life?	1=YES<br>2=NO<br>96=PREFER NOT TO ANSWER	If No or prefer not to answer, skip to Q26
Q25	Among those that died in the first year of life, how many died in the first month	__________(NUMBER)	

ANC, DELIVERY AND PNC, CHILD HEALTH			
Now I would like to ask some questions about your use of maternal and newborn services.			
	**QUESTIONS**	**CODING**	**SKIP/NOTES**
Q26	Are you here for post-delivery discharge, postnatal care, or a child health visit?	1=DELIVERY<br>2=POSTNATAL CARE<br>3=CHILD HEALTH VISIT	
Q27	How many facility-based antenatal visits did you attend for your most recent pregnancy?<br><br>IF NONE, CODE ZERO	_______NUMBER OF VISITS<br>96=DON'T REMEMBER	If zero, skip to 29
Q28	How many months pregnant were you when you first received antenatal care for your recent pregnancy?	________NUMBER OF MONTHS	
Q29	Where did you deliver your child?	1 = AT THIS FACILITY<br>2 = ANOTHER HEALTH FACILITY<br>3 = AT HOME<br>4 = OTHER___________<br>(SPECIFY)	
Q30	Who delivered your baby?<br><br>PROBE. CIRCLE ALL MENTIONED.	A=DOCTOR<br>B=MIDWIFE/NURSE<br>C=OTHER HEALTH WORKER<br>D=TRADITIONAL BIRTH ATTENDANT<br>E=FAMILY MEMBER<br>F=NO ONE<br>G=NOT SURE<br>X=OTHER___________<br>(SPECIFY)	
Q31	Did you use a Maternity Waiting Home for this pregnancy and/or post-delivery?	1=YES<br>2=NO	2→Q43 FILTER
Q32	What is the name of the Maternity Waiting Home?	1=AREA 25<br>2=KASUNGU<br>3=OTHER _____________<br>(SPECIFY)	
Q33	How many months pregnant were you when you first came to the Maternity Waiting Home?	_________ (NUMBER OF MONTHS)<br>88 (CIRCLE 88 IF POST DELIVERY)	
Q34	Who decided that you should seek care at a Maternity Waiting Home?<br><br>PROBE: CIRCLE ALL MENTIONED.	A=RESPONDENT<br>B=SPOUSE<br>C=MOTHER-IN-LAW<br>D=OTHER FAMILY MEMBER<br>E=HEALTH WORKER<br>F=TRADITIONAL BIRTH ATTENDANT<br>G=TRADITIONAL HEALER<br>H=OTHER___________<br>(SPECIFY)	
Q35	How did you learn about the Maternity Waiting Home?<br><br>PROBE: CIRCLE ALL MENTIONED.	A=OUTREACH EVENT<br>B=HEALTH WORKER<br>C=RADIO<br>D=TELEVISION<br>E=TRIBAL AUTHORITY LEADER/CHIEF<br>F=TRADITIONAL BIRTH ATTENDANT<br>G=TRADITIONAL HEALER<br>H=FRIEND/FAMILY MEMBER<br>I=OTHER__________________<br>(SPECIFY)	
Q36	What were the reasons for seeking care at a Maternity Waiting Home?<br><br>PROBE: CIRCLE ALL MENTIONED	A=FOR A GOOD PREGNANCY OUTCOME<br>B=COMPLICATIONS<br>C=PREVIOUS USE OF A MATERNITY WAITING HOME<br>D=REFERRAL FROM A HEALTH FACILITY OR A HEALTH WORKER<br>E=RECOMMENDED BY A FAMILY MEMBER OR FRIEND	

		F=OTHER___________<br>(SPECIFY)	
Q37	While at the Maternity Waiting Home for the most recent pregnancy, did you attend any educational sessions?	1=YES<br>2=NO<br>96=DON'T KNOW/DON'T REMEMBER	 2→Q40<br>96→Q40
Q38	Which educational sessions did you attend?<br><br>PROBE. CIRCLE ALL MENTIONED	A=HEALTH EDUCATION<br>B=NUTRITIONAL EDUCATION<br>C=HOME CRAFT (PREPARATION OF FOODS)<br>D=OTHER __________________<br>(SPECIFY)	
Q39	What topics were covered during the training?<br><br>PROBE. CIRCLE ALL MENTIONED	A=DANGER SIGNS IN PREGNANCY<br>B=PERSONAL AND ENVIRONMENTAL HYGIENE<br>C=NUTRITION IN PREGNANCY and POSTNATAL<br>D=POSTPARTUM FAMILY PLANNING<br>E=BIRTH PREPAREDNESS<br>F=PREVENTION OF MOTHER TO CHILD TRANSMISSION OF HIV<br>G=NEONATAL CARE<br>H=IMMUNISATIONS (MOTHER AND BABY)<br>I=EXCLUSIVE BREASTFEEDING<br>J=POSTNATAL CARE<br>K=DANGER SIGNS POST DELIVERY (MOTHER, BABY)<br>L=HEALTHY LIVING THROUGH ACTIVITIES (SEWING,<br>M=GARDENING/PERMACULTURE, EXERCISE)<br>N=MALE CHAMPIONSHIP OF HEALTH<br>O=INFECTIONS IN PREGNANCY (including HIV)<br>P=OTHER________________________<br>X=DON'T REMEMBER	
Q40	While at the Maternity Waiting Home, did you receive antenatal care?	1=YES<br>2=NO<br>96=DON'T KNOW/DON'T REMEMBER	 2→Q42a<br>96→Q42a
Q41	How many antenatal care visits did you receive while at the Maternity Waiting Home?	_______NUMBER OF VISITS<br>96= don't remember	
Q42a	Would you recommend this Maternal Waiting Home to your friends and family?	1=YES<br>2=NO	
Q42b	How satisfied were you with the quality of services at the Maternity Waiting Home (very satisfied, satisfied, neutral, unsatisfied, very unsatisfied)?	5=VERY SATISFIED<br>4=SATISFIED<br>3=NEUTRAL<br>2=UNSATISFIED<br>1=VERY UNSATISFIED	
Q42c	How satisfied were you with the quality of educational messages at the Maternity Waiting Home (very satisfied, satisfied, neutral, unsatisfied, very unsatisfied)?	5=VERY SATISFIED<br>4=SATISFIED<br>3=NEUTRAL<br>2=UNSATISFIED<br>1=VERY UNSATISFIED	ALL GO TO Q44
Q43a	FOR WOMAN WHO DID NOT DELIVER IN A HEALTH FACILITY ONLY (Q29=3, 4 OR OTHER)<br>Why did you not deliver your baby in a health facility?<br><br>PROBE. CIRCLE ALL MENTIONED.	A=DISTANCE<br>B=LACK OF KNOWLEDGE<br>C=DIDN"T FEEL IT WAS NEEDED<br>D=LACK OF TRANSPORTATION<br>E=PERMISSION NOT GRANTED<br>F=COSTS<br>G=PERCEPTION OF QUALITY<br>H=PERCEPTION OF HEALTH WORKER ATTITUDES<br>I=PERCEPTION OF HEALTH WORKER AVAILABILITY<br>J=MIDWIFE NOT AVAILABLE<br>K=DOCTOR NOT AVAILABLE<br>L=CROWDING/WAITING TIME<br>M=FEAR OF FACILITIES<br>N=AVAILABILITY OF A TRAINED TBA<br>O=DELIVERED ON THE WAY TO THE FACILITY	

		X=OTHER________________________<br>(SPECIFY)	
Q43b	FOR WOMAN WHO DID NOT USE A MATERNITY WAITING HOME ONLY (Q31=2)Did you know that this facility had a Maternity Waiting Home before you came here today?	1=YES<br>2=NO<br>3= DOESN'T KNOW WHAT A MATERNITY WAITING HOME IS	
Q44	Have you used a Maternity Waiting Home for a prior pregnancy?	1=YES<br>2=NO<br>3=FIRST PREGNANCY	
Q45	During your most recent pregnancy were you told about possible pregnancy complications?	1=YES<br>2=NO<br>96=DON'T KNOW/DON'T REMEMBER	 2→Q48<br>96→Q48
Q46	What were the complications you were told about?<br><br>PROBE. CIRCLE ALL MENTIONED.	A=BLEEDING<br>B=FEVER<br>C=SWOLLEN HANDS/FACE/FEET<br>D=TIREDNESS/BREATHLESSNESS<br>E=HEADACHE/BLURRED VISION<br>F=CONVULSIONS<br>G=PROLONGED LABOR<br>H=HIV INFECTIONS<br>I=ANEMIA<br>J=OTHER___________________<br>(SPECIFY)	
Q47	Where did you get information on possible pregnancy complications?<br><br>PROBE. CIRCLE ALL MENTIONED	A=MATERNITY WAITING HOME<br>B=HEALTH FACILITY<br>C=HEALTH WORKER<br>D=TRADITIONAL BIRTH ATTENDANT<br>E=FAMILY MEMBER/FRIEND<br>F=OTHER___________________<br>(SPECIFY)	
Q48	Were you told that you might be at high risk for a pregnancy complication?	1=YES<br>2=NO	
Q49	Did you experience any pregnancy related complications for your most recent pregnancy?	1=YES<br>2=NO	
Q50	During antenatal care were you told about newborn complications?	1=YES<br>2=NO<br>96=DON'T KNOW/DON'T REMEMBER<br>98=NO ANTENTAL CARE	 2→Q53<br>96→Q53<br>98→Q53

Q51	What were the complications you were told about?<br><br>PROBE: CIRCLE ALL MENTIONED.	A=FEVER<br>B=DIFFICULTY BREATHING<br>C=COUGHING<br>D=DIARRHEA<br>E=JAUNDICE<br>F=INABILITY TO FEED<br>G=UMBLICAL CORD INFECTION<br>H=PRETERM/EARLY BIRTH<br>I=OTHER___________________<br>(SPECIFY)	
Q52	Where did you get information on possible newborn complications?<br><br>PROBE. CIRCLE ALL MENTIONED.	A=MATERNITY WAITING HOME<br>B=HEALTH FACILITY<br>C=HEALTH WORKER<br>D=TRADITIONAL BIRTH ATTENDANT<br>E=FAMILY MEMBER/FRIEND<br>F=OTHER___________________<br>(SPECIFY)	
Q53	Did your newborn experience any complications during delivery or shortly afterwards?	1=YES<br>2=NO	
Q54	During pregnancy or after delivery were you given any health messages about breastfeeding?	1=YES<br>2=NO<br>3=DON'T KNOW/DON'T REMEMBER	
Q55	What are some things you can do to take care of yourself after delivery?<br><br>PROBE. CIRCLE ALL MENTIONED.	A=EAT WELL<br>B=REST<br>C=BREASTFEED THE BABY<br>D=WATCH FOR SIGNS OF COMPLICATIONS<br>F=OTHER___________________<br>(SPECIFY)	
Q56	What are some important practices for taking care of a newborn?<br><br>PROBE. CIRCLE ALL MENTIONED.	A=KEEPING THE BABY WARM<br>B=CLEAN CORD CARE<br>C=WATCHING FOR SIGNS OF SICKNESS/COMPLICATIONS<br>D=POSTNATAL CHECKS<br>E=IMMUNIZATIONS<br>F=SAFE DISPOSAL OF FECES<br>G=WASHING HANDS<br>H=BREASTFEEDING<br>I=OTHER___________________<br>(SPECIFY)	
Q57	When should breastfeeding be initiated?	1=IMMEDIATELY/WITHIN AN HOUR OF BIRTH<br>2=WITHIN A DAY<br>3=WITHIN A FEW DAYS<br>4=OTHER___________________<br>(SPECIFY)	
Q58	How long should a baby be exclusively breastfed?	1=NOT AT ALL<br>2=FOR A FEW DAYS<br>3=FOR LESS THAN 6 MONTHS<br>4=FOR 6 MONTHS<br>5=FOR MORE THAN 6 MONTHS<br>4=OTHER___________________<br>(SPECIFY)	
Q59	Can HIV be transmitted from mother-to-child?	1=YES<br>2=NO<br>96=DON'T KNOW/DON'T REMEMBER	2→Q62<br>96→Q62
Q60	In what ways can HIV be transmitted from mother-to-child?<br><br>PROBE. CIRCLE ALL MENTIONED.	A=DURING PREGNANCY<br>B=DURING DELIVERY<br>C=WHILE BREASTFEEDING<br>D=DON"T KNOW	
Q61	Are there medicines that can help prevent a baby whose mother is HIV-positive from getting HIV?	1=YES<br>2=NO<br>3=DON'T KNOW/DON'T REMEMBER	
Q62	For the health of the mother and baby what is a good interval (in months) between the birth of children?	1=LESS THAN 1 YEAR<br>2=2 YEARS<br>3=3 YEARS<br>4=4-5 YEARS	

		5=MORE THAN 5 YEARS<br>6=DON'T KNOW/REMEMBER<br>7=OTHER___________________<br>(SPECIFY)	
Q63	In your opinion how important are immunizations for your children (very important, important, neutral, unimportant, very unimportant)?	5=VERY IMPORTANT<br>4=IMPORTANT<br>3=NEUTRAL<br>2=UNIMPORTANT<br>1=VERY UNIMPORTANT	
Q64	In your opinion how important are postnatal checks for yourself (very important, important, neutral, unimportant, very unimportant)?	5=VERY IMPORTANT<br>4=IMPORTANT<br>3=NEUTRAL<br>2=UNIMPORTANT<br>1=VERY UNIMPORTANT	
Q65	In your opinion how important are postnatal checks for your baby (very important, important, neutral, unimportant, very unimportant)?	5=VERY IMPORTANT<br>4=IMPORTANT<br>3=NEUTRAL<br>2=UNIMPORTANT<br>1=VERY UNIMPORTANT	
Q66	In your opinion how important is it for a woman to deliver her baby in a health facility (very important, important, neutral, unimportant, very unimportant)?	5=VERY IMPORTANT<br>4=IMPORTANT<br>3=NEUTRAL<br>2=UNIMPORTANT<br>1=VERY UNIMPORTANT	
Q67a	Have you initiated breastfeeding	1=YES<br>2=NO<br>3=HAD A STILLBIRTH/MISCARRIAGE	 2→Q68a<br>3→Q68a
Q67b	When did your baby first start breastfeeding	1=IMMEDIATELY AFTER BIRTH<br>2=WITHIN THE FIRST HOUR AFTER BIRTH<br>3=WITHIN THE FIRST DAY AFTER BIRTH<br>4=OTHER _________________<br>(SPECIFY)	
Q68a	Before you became pregnant with your most recent pregnancy, did you ever use a family planning method prior to your most recent pregnancy?	1=YES<br>2=NO	 2→Q69
Q68b	What method did you ever use prior to getting pregnant?<br><br>CIRCLE ALL MENTIONED	A=NATURAL FAMILY PLANNING/WITHDRAWAL<br>B=BREASTFEEDING<br>C=MALE CONDOM<br>D=FEMALE CONDOM<br>E=ORAL CONTRACEPTIVES<br>F=EMERGENCY CONTRACEPTIVE PILL<br>G=INJECTION<br>H=IMPLANT<br>I=IUD/LOOP<br>J=MALE STERILIZATION<br>K=FEMALE STERILIZATION<br>L=OTHER___________________<br>(SPECIFY)	
Q69	Were you counselled on family planning during your most recent pregnancy or after your most recent delivery?	1=YES<br>2=NO<br>3=DON'T KNOW/DON'T REMEMBER	 2→71<br>3→71
Q70	What family planning methods were you counselled on?<br><br>PROBE. CIRCLE ALL MENTIONED.	A=NATURAL FAMILY PLANNING/WITHDRAWAL<br>B=BREASTFEEDING<br>C=MALE CONDOM<br>D=FEMALE CONDOM<br>E=ORAL CONTRACEPTIVES<br>F=EMERGENCY CONTRACEPTIVE PILL<br>G=INJECTION<br>H=IMPLANT<br>I=IUD<br>J=MALE STERILIZATION	

		K=FEMALE STERILIZATION<br>L=OTHER____________________<br>(SPECIFY)	
Q71	In your opinion should men be involved in maternal health care?	1=YES<br>2=NO<br>3=DON'T KNOW	
Q72	In your opinion should men be involved in newborn health care?	1=YES<br>2=NO<br>3=DON'T KNOW	
Q73	Did your community hold any events to promote the use of maternal health care services?	1=YES<br>2=NO	2→Q76
Q74	Who conducted the events?<br><br>PROBE. CIRCLE ALL MENTIONED.	A=TRIBAL AUTHORITY LEADER/CHIEF<br>B=VILLAGE ELDERS<br>C=HEALTH WORKERS<br>D=TRADITIONAL BIRTH ATTENDANTS<br>E=WOMEN'S GROUPS<br>F=SAFE MOTHERHOOD REPRESENTATIVE<br>G=MAI MWANA<br>H=FPAM<br>I=UNFPA<br>J=OTHER NGO<br>K=UNC/LILONGWE MEDICAL TRUST<br>L=MAIKHANDA<br>X=OTHER____________________<br>(SPECIFY)	
Q75	Did you attend any of the events?	1=YES<br>2=NO	
FILTER 1 – THIS SECTION Q76 to Q89b IS FOR WOMEN WITH A RECENT DELIVERY OR A RECENT POSTNATAL CHECK ONLY (SKIP to Q90 FOR WOMEN BRINGING A BABY FOR A WELLNESS CHECK			
Q76	For how long after delivery did you stay in the Maternity Waiting Home?	0 MARK ZERO IF SHE DID NOT USE THE MWH OR IF SHE DELIVERED AT HOME AND NEVER USED MWH<br><br>_____(NUMBER OF HOURS. IF MORE THAN 1 DAY CONVERT TO HOURS)	
Q77	Did someone check on your health before you were discharged?	1=YES<br>2=NO<br>96=DID NOT DELIVER IN A FACILITY	2→Q80<br>96→Q85a
Q78	Was your temperature taken before discharge?	1=YES<br>2=NO	
Q79	Were you checked for bleeding before discharge?	1=YES<br>2=NO	
Q80	Did someone check on your baby before you were discharged?	1=YES<br>2=NO<br>3=STILLBIRTH/MISCARRIAGE	2→Q83<br>3→Q87
Q81	Was the baby's cord checked?	1=YES<br>2=NO	
Q82	Was the baby's temperature taken?	1=YES<br>2=NO	
Q83	Was the baby weighed?	1=YES<br>2=NO	
Q84	Were you taught how to keep the baby warm?	1=YES<br>2=NO	
Q85a	Do you intend to breastfeed your baby exclusively, that is to only provide your baby with breast milk?	1=YES<br>2=NO<br>96=DON'T KNOW	2→Q86<br>96→Q86
Q85b	For how long do you intend to exclusively breastfeed your baby?	________(NUMBER OF MONTHS)	FOR ALL RESPONSES GO TO Q87
Q86	Do you intend to breastfeed your baby at all?	1=YES<br>2=NO<br>96=DON'T KNOW	
Q87	Are you currently using a family planning method?	1=YES<br>2=NO	2→Q89a

Q88	Which family planning method are you using?	A=NATURAL FAMILY PLANNING/WITHDRAWAL<br>B=BREASTFEEDING<br>C=MALE CONDOM<br>D=FEMALE CONDOM<br>E=ORAL CONTRACEPTIVES<br>F=EMERGENCY CONTRACEPTIVE PILL<br>G=INJECTION<br>H=IMPLANT<br>I=IUD<br>J=MALE STERILIZATION<br>K=FEMALE STERILIZATION<br>L=OTHER____________________<br>(SPECIFY)	FOR ALL RESPONSES END INTERVIEW
Q89a	Do you plan to use a family planning method in the next six months?	1=YES<br>2=NO<br>3=DON'T KNOW	IF YES OR DON'T KNOW, END INTERVIEW AND THANK RESPONDENT
Q89b	If you would not consider using a family planning method, why not?<br><br>PROBE. CIRCLE ALL MENTIONED.	A=WOMEN GAIN WEIGHT<br>B = WOMEN BECOME STERILE<br>C = MEN DON'T LIKE WOMEN USING CONTRACEPTIVES<br>D= FEAR OF SIDE EFFECTS<br>E= OTHER ________________________<br>(SPECIFY)	FOR ALL RESPONSES END INTERVIEW AND THANK RESPONDENT
FILTER 2 – Q90 to Q104b ARE FOR WOMEN BRINGING A CHILD FOR A WELLNESS CHECK ONLY			
Q90	How old is your baby?	_______MONTHS _____DAYS<br><br>(IF LESS THAN ONE MONTH RECORD IN DAYS. CIRCLE MONTHS OR DAYS)	
Q91	Has your child been very sick since birth?	1=YES<br>2=NO	2→Q93
Q92	Did you bring your child to a health facility when he or she was sick?	1=YES<br>2=NO	
Q93	Did your baby have a health check within the first two days of life?	1=YES<br>2=NO	
Q94	Have you breastfed your baby exclusively, that is to only provide your baby with breast milk?	1=YES<br>2=NO<br>96=DON'T KNOW	2→Q98<br>96→Q98
Q95	For how long have you exclusively breastfed your baby?	NUMBER OF WEEKS ________________<br>(IF SINCE BIRTH, INDICATE CHILD'S AGE IN WEEKS)	
Q96	Are you still exclusively breastfeeding your baby?	1=YES<br>2=NO	2→Q100
Q97	For how long do you intend to exclusively breastfeed your baby?	___________(NUMBER OF MONTHS)	FOR ALL RESPONSES SKIP TO Q100
Q98	Have you been breastfeeding your child	1=YES<br>2=NO	2→Q100
Q99	For how long do you intend to breastfeed your baby?	___________(NUMBER OF MONTHS)	
Q100	Has your child received any immunizations?	1=YES<br>2=NO	1→Q102
Q101	Do you plan to have your child immunized?	1=YES<br>2=NO	
Q102	Are you currently using a family planning method?	1=YES<br>2=NO	2→Q104
Q103	Which family planning method are you using?	A=NATURAL FAMILY PLANNING/WITHDRAWAL<br>B=BREASTFEEDING<br>C=MALE CONDOM	FOR ALL RESPONSES END INTERVIEW

		D=FEMALE CONDOM<br>E=ORAL CONTRACEPTIVES<br>F=EMERGENCY CONTRACEPTIVE PILL<br>G=INJECTION<br>H=IMPLANT<br>I=IUD<br>J=MALE STERILIZATION<br>K=FEMALE STERILIZATION<br>L=OTHER____________________<br>(SPECIFY)	
Q104a	Do you plan to use a family planning method in the next six months?	1=YES<br>2=NO<br>3=DON'T KNOW	IF YES OR DON'T KNOW, END INTERVIEW
Q104b	If you would not consider using a family planning method, why not?<br><br>PROBE. CIRCLE ALL MENTIONED.	A=WOMEN GAIN WEIGHT<br>B = WOMEN BECOME STERILE<br>C = MEN DON'T LIKE WOMEN USING CONTRACEPTIVES<br>D= FEAR OF SIDE EFFECTS<br>E= OTHER ________________________<br>(SPECIFY)	

Thank you. We very much appreciate your time. Your responses will help us improve maternal and newborn care in your area.
