# Supplementary material for: Evaluation of a maternity waiting home and community education program in two districts of Malawi
Source: BMC Pregnancy Childbirth. 2018 Nov 23;18:457. doi: 10.1186/s12884-018-2084-7 (PMC6251123; doi:10.1186/s12884-018-2084-7)
Supplement: Supplementary file 2 — Discharge Questionnaire. (PDF 201 kb) [file 12884_2018_2084_MOESM2_ESM.pdf]

MALAWI MATERNITY WAITING HOME DISCHARGE FORM

Malawi Waiting Home Discharge Instructions:

1. Forms are administered in person by the Maternity Waiting Home Advocate in a private and quiet location.
2. Discharge form is to help assess maternity waiting home services in order to improve them, so please complete when a patient is leaving the maternity waiting home.

DISCHARGE TOOL	
**1.**	**INTERVIEW INFORMATION**
	A. INTERVIEWER NAME: ____<br>B. DATE OF INTERVIEW: ___/____/_____ (dd / mm / yyyy)<br>C. MATERNITY WAITING HOME (circle one):<br>Kasungu 1<br>Area 25 2
**2.**	**RESPONDENT IDENTIFICATION**
	Maternity Waiting Home Registration Number [ ][ ][ ][ ][ ]

SECTION 1: ABOUT YOU AND GETTING TO THE MATERNITY WAITING HOME

The following questions ask about your background, how you got to the Maternity Waiting Home, why you came and who came with you.

#	Question	Response Options	Go To
**1**	*Muli ndi zaka zingati?*<br>What is your age?	Age in years [__|__]<br>Don't know 88<br>No Response 99	
**2**	*Mumakhala kuti?*<br>Where do you live?<br>*(Mudzi ndi mfumu yaikulu) Village Name & TA (write in)*	Village___________________<br>TA ____________________<br>No Response 99	
**3**	*Moyo wanu uli bwanji?*<br>In general, how would you rate your overall health?	Poor 1<br>Good 2<br>Very good 3<br>Don't know 88<br>No Response 99	
**4**	*Amene anakudikirirani ndani pamene munali kuchiyembekezero kuno?*<br>Who was your guardian while you were staying at the Waiting Home? (circle all that apply)	Mother 1<br>Mother-in-law 2<br>Sister 3<br>Husband 4<br>Friend 5<br>Child 6<br>No one 7<br>_____________Other 8	
**5**	*Kodi mwakhala nthawi yaitali bwanji kunyumba yodikirai musanachire?*<br>How long were you here at maternity waiting home before delivery/departure?	Less than 1 day 1<br>1-7 days 2<br>1-2 weeks 3<br>3-4 weeks 4<br>More than 4 weeks 5<br>Don't know 88	

Maternity Waiting Home Patient Register Number

**SECTION 2: THIS PREGNANCY**<br>*The following questions ask information about your current pregnancy.*			
**#**	**Question**	**Response Options**	**Go To**
**6**	*Kodi m'mene mumabwera kuno kudzadikilira munali ndi miyezi ingati?*<br>How far along were you in this pregnancy when you arrived at the maternity waiting home?	Almost at due date 1<br>1 month from due date 2<br>2-3 months from delivery 3<br>Half through pregnancy 4<br>N/A: already delivered 5<br>Don't know 88	**If pregnant →Q8**
**7a**	*Ngati mwabereka kale. Kodi munachira liti?*If you have delivered already, how long ago did you give birth?	Less than 1 day 1<br>1-2 days 2<br>Other 3	
**7b**	*Ngati mwabereka kale, kodi zimene mukachite ndi chiani kuti mwana wanu akhale ndi moyo wathanzi?*<br>If you have already delivered, what are you going to do to keep your baby in good health	Keep the baby warm 1<br>Exclusively breast feed the baby 2<br>Keep the cord stump clean 3<br>Take the baby to u/5 clinic for immunization & growth monitoring 4	
**8**	*Kodi ndi chifukwa chanji mwana akabadwa amalandira katemera?*<br>Why should a new born receive immunization?	Don't know 1<br>To be protected from infectious diseases 2	
**9a**	*Kodi ndi matenda ati amene mwana angatetezedwe ndi katemera?*<br>What diseases can your baby be protected from by immunization? Circle all that are mentioned	Measles 1<br>TB 2<br>Polio 3<br>Tetanus 4<br>Pneumonia 5<br>Whooping cough 6	
**9b**	*Kodi pali katemera woteteza amayi?*<br>*Ngati alipo, katemera ameneyi ndi wachiani?*<br>Are there any immunizations for the mother?<br>If yes what are they?	Yes 1<br>No 2<br><br>Tetanus 1<br>Others 2	
**10**	*Kodi zimene mungapange ndi chiani kuti inu muzisamalire?*<br>What can you do to care for yourself after delivery? (Circle all that are mentioned)	Take adequate rest 1<br>Eat nutritious diet 2<br>Observe amount of vaginal bleeding 3<br>Keep fit through exercise 4<br>Maintain good personal & environmental hygiene 5<br>Report to HC if danger signs develop (such as fever, bleeding, heavy offensive vaginal discharge, blurred vision, headache) 6	
**11**	*Kodi mayi angapange chiani kuti atsamupatsire mwana wache kachirombo?*<br>How can transmission of HIV from mother to child be prevented? (Circle all that are mentioned)	Getting tested at the beginning of ANC 1<br>Encourage partner/husband to be tested as well 2<br>Taking ARV if tested positive 3<br>Baby to receive ARV after delivery 4<br>Exclusive breast feeding for at least 6 weeks 5<br>Retesting at regular interval & avoid reinfection 6	
**12**	*Kodi mumakonzekera bwanji mukamadikira kubadwitsa mwana?*<br>What can you do to prepare for delivery? (Circle all that are mentioned)	Keep delivery bag ready with baby & mum's clothes 1<br>Discuss & organize transport with husband/partner 2<br>Prepare to wait at a MWH if health facility is far 3	
**13**	*Kodi kufunika koyamwitsa mwana bele ndi chiani?*<br>What is the importance of breast feeding? (Circle all that are mentioned)	Breast milk is the best food for baby 1<br>Breast milk contains first immunity for baby 2<br>Breast milk is always ready, doesn't need preparation 3<br>Breastfed babies don't suffer from diarrhea frequently 4<br>Breastmilk is cheap 5<br>Breastfeeding helps mother and baby to bond 6	

**14 a**	*Kodi woyamwitsa anayenera kudya zakudya zamtundu wanji?*<br>What consists of a well-balanced diet for pregnancy and lactating woman?<br>(Circle all that are mentioned)	Fish, beef, chicken 1<br>Fruits and juice 2<br>Vegetables 3<br>Water 4<br>Potatoes, maize, flour 5<br>Legumes 6	
**14 b**	*Kodi ndi chifukwa chiani mayi woyamwitsa amayenera kudya zakudya za magulu?* Why is it important for a pregnant and a lactating mother to eat a well-balanced diet?	To get adequate nutrients for body functions 1<br>To maintain adequate blood level 2<br>To support immune functions 3	
**15**	*Kodi mumapanga bwanji kuti pakhomo panu pakhale paukhondo?*<br>How do you maintain/keep personal and environmental hygiene?<br>(Circle all that are mentioned)	Daily bath 1<br>Keep clothes clean 2<br>Change sanitary pads/towels frequently 3<br>Keep house clean & free from cockroaches/mosquitoes 4<br>Keep grounds clean 5<br>Encourage use of a pit for waste disposal 6	

**SECTION 3: EDUCATION AND KNOWLEDGE**			
**#**	**Question**	**Response Options**	**Go To**
**16**	Did you attend any educational sessions at the Waiting Home?<br>*Kodi manapanga nawo maphunziro ali onse panthawi imene munali kunyumba yodikirira ija?*	Yes 1<br>No 2<br>Don't know 88<br>No Response 99	**"No"→Q20**
**17**	*Nanga ndi maphunziro anji amene munapanga nawo?*<br>Which educational sessions did you attend at the Waiting Home? (Check all that apply)	Family planning 1<br>Nutrition 2<br>Breastfeeding 3<br>HIV Prevention, Care 4<br>Baby care 5<br>Postpartum care 6<br>Danger signs in pregnancy 7<br>Danger signs in post-delivery 8<br>Personal Hygiene 9<br>Environmental hygiene 10<br>Birth readiness 11<br>Immunizations 12<br>Healthy living through activities 13<br>Male championship 14<br>Infections in pregnancy 15<br>Permaculture16<br>Arts and crafts17<br>__________Other 18<br>Don't remember 88<br>No Response 99	
**18**	*Kodi mungatchule mitu itatu imene mwaphunzira zinthu zokuthandizani?*<br>Which 3 topics did you learn the most from?	______________________<br>______________________<br>______________________	
**19**	*Nanga pali zinthu zimene inuyo munakonda mutaphunzirapo?*<br>What other topics would you have liked to be taught?	______________________<br>______________________<br>______________________	

**20**	*Kodi ndi njira ziti zakulera zimene mukudziziwa ?*<br>What modern family planning method(s) do you know? (Circle all that are mentioned)	None 1<br>Natural family planning 2<br>Withdrawal 3<br>Breastfeeding 4<br>Male condom 5<br>Female condom 6<br>Oral contraceptive pill 7<br>Emergency contraceptive pill 8<br>Injection 9<br>Implant 10<br>IUCD 11<br>Tubal sterilization 12<br>Vasectomy 13<br>_______________Other 14<br>Don't know 88	
**21**	*Kodi munaphunzira kuti za njira zakulera zamakono?(Chongani zonse zimene atchula)*<br>Where did you learn about "Modern Family Planning"? (Circle all that apply)	Here at the MWH 1<br>Another clinic 2<br>Family member/friend 3<br>Village leader 4<br>_______________Other 7<br>Don't remember 88	
**22**	*Kodi mmene mwabelekamu, mugwiritsa njira iri yonse yolera?*<br>Following this delivery, are you planning to use any family planning method?	Yes 1<br>No 2<br>Don't know 88<br>No Response 99	 **"No"→Q24**<br>**"88"→Q24**<br>**"99"→Q24**
**23**	*Nanga njira imene mukufuna kugwiritso nchito ndi iti?*<br>If yes, which method will you use?	None 1<br>Natural family planning 2<br>Withdrawal 3<br>Breastfeeding 4<br>Male condom 5<br>Female condom 6<br>Oral contraceptive pill 7<br>Emergency contraceptive pill 8<br>Injection 9<br>Implant 10<br>IUCD 11<br>Tubal sterilization 12<br>Vasectomy 13<br>_________Other 14<br>Don't know 88	**All responses go to Q25**
**24**	*Nanga ndi chifukwa chiani simukufuna kutenga njira yolera pano?*<br>If you would not consider using a modern family planning method, why not?	Women gain weight 1<br>Women become sterile 2<br>Men don't like women who use contraceptives 3<br>Fear of side effects 4<br>Other 5<br>Don't know 6	

Maternity Waiting Home Patient Register Number | | | | | |

SECTION 4: GENERAL PREGNANCY KNOWLEDGE

#	Question	Response Options	Go To
25	*Kodi mukuganiza kuti mayi wapakati ayendere sikelo kangati kuti alandire chithandizo chokwanira?*<br>In your opinion, how many antenatal/prenatal care visits should a pregnant woman get in order to get adequate care throughout pregnancy?	None 1<br>One 2<br>Two 3<br>Three 4<br>Four 5<br>More than four 6<br>______________Other 7<br>Don't know 88<br>No Response 99	
26	*Nanga ndi msinkhu wabwino woti mzimayi ayambe kubereka ndi zaka zingati?*<br>What is the best age for a woman to start bearing children?	*Below 16 years 1*<br>*17 to 19 years 2*<br>*20 and above 3*<br>*Other number (write in) 4*<br>[__|__]<br>Don't know 88<br>No Response 99	

SECTION 5: PERMACULTURE & HOME CRAFT KNOWLEDGE

#	Question	Response Options	Go To
27	*Kodi mungapange bwanji kuti mukhale ndi ndiwo zamasamba ndi zipatso pakhomo panu?*<br>What can you do to ensure you have vegetables, fruits in your household? (Circle all that are mentioned)	Create a garden from pieces of land within their household 1<br>Use organic manure 2<br>Recycle used water at home for watering garden 3<br>Other 4	
28	*Kodi chimene mungapange ndi chiyani kuti muzipeza ndalama zokuthandizirani pakhomo?*<br>What income generating activities have you had training in? (Circle all that apply)	Small businesses e.g. selling vegetables and farm produce 1<br>Getting small loans 2<br>Producing arts and crafts for sale 3<br>Others 4	

SECTION 6: PLEASE RATE THE FOLLOWING ASPECTS OF THE MATERNITY WAITING HOME:

		Response options					
Question: Please rate the following:		Poor 1	Fair 2	Good 3	Very Good 4	Excellent 5	Don't Know 88
29	*Kodi malo amene pali nyumba yodikirayi pali bwanji?*<br>The location of the Maternity Waiting Home within the health facility grounds	O	O	O	O	O	O
30	*Kodi masana panyumayi phokoso linali bwanji?*<br>The noise level during the day	O	O	O	O	O	O
31	*Nanga nthawi yausiku, phokoso linali bwanji?*<br>The noise level at night	O	O	O	O	O	O
32	*Nanga mumawala bwanji?*<br>The amount of light	O	O	O	O	O	O
33	*Nanga madzi ophikira ndi akumwa ndi wokwanira bwanji?*<br>The **amount** of water for drinking/cooking	O	O	O	O	O	O
34	*Nanga madzi osamba ndi wokwanira bwanji?*	O	O	O	O	O	O

	The **amount** of water for bathing						
35	*Nanga ukhondo wa ku zimbuzi uli bwanji?*<br>The cleanliness of the toilets	o	o	o	o	o	o
36	*Nanga malo ophikira ali bwino bwanji?*<br>The kitchen/food preparation facilities	o	o	o	o	o	o
37	*Nanga pamalo ndi potetezeka bwanji?*<br>Your sense of personal safety/security.	o	o	o	o	o	o

SECTION 7: OVERALL RATINGS AND RECOMMENDATIONS		
**#**	**Question**	**Response Options**
**38**	*Kodi mmene mwakhalira mnyumba yodikirirayi, mugakauze anzanu kuti naowonso azapange chidikiriro konkuno?*<br>Would you recommend the Waiting Home to your friends or family?	Yes 1<br>No 2<br>Don't know 88
**39**	*Nanga mukazakhala ndi pakati pena, mungazapangenso chidikiriro pompano?*<br>Would you use the Waiting Home again if needed?	Yes 1<br>No 2<br>Don't know 88
**40**	*Kodi pali chimene chingakonzedwe kuti amai adzikhala mokondwera panyumba yodikirirayi?*<br>How could the maternity waiting home be improved?	Open response (write in)
**41**	*Nanga pali zina zimene mungatiudziwitse kuti tikonze panyuma yodikirirayi?*<br>What other things should we know about the Waiting Home that would help us make it better?	Open response (write in)
